# Navigating University Openness in Research Policy Inconsistent with Indigenous Data Sovereignty: *A Case Analysis*

**DOI:** 10.1002/eahr.500202

**Published:** 2024

**Authors:** Molly Wick, Deanna Erickson, Joel Hoffman, Lucinda Johnson, Ted Angradi

**Affiliations:** PhD candidate at the University of Minnesota Duluth and a student trainee at the U.S. Environmental Protection Agency Great Lakes Toxicology and Ecology Division; Director of the Lake Superior National Estuarine Research Reserve; manager of the Ecosystem Services Branch at the U.S. Environmental Protection Agency Great Lakes Toxicology and Ecology Division; senior research fellow at the University of Minnesota Natural Resources Research Institute; retired research biologist for the U.S. Environmental Protection Agency Great Lakes Toxicology and Ecology Division

**Keywords:** Indigenous communities, research sovereignty, data sovereignty, open research policies, institutional review boards

## Abstract

Indigenous nations and communities in the United States have rights as sovereign governments to exercise control and ownership over all data and information generated by or from the tribes, tribal members, or tribal resources. Indigenous nations exercise these rights through data ownership policies established in response to unethical research practices in research involving Indigenous communities. Most universities in the U.S. have “openness in research” policies to ensure academic freedom to publish freely, exercised by retaining university control of data. Here, we describe our study of cultural ecosystem services in the St. Louis River estuary region (Nagaajiwanaang in the language Ojibwemowin) in Duluth, Minnesota, and Superior, Wisconsin, U.S., an area that includes portions of the 1854 and 1842 Ceded Territories and reservation lands of a local band of Ojibwe (hereafter referred to as “the Band”). In this university-led, Band-supported study, both the university and the Band sought ownership of data collected based on their respective policies, resulting in a research delay of nearly a year. We found that open research policies that do not consider Indigenous sovereignty can hamper collaboration between university researchers and tribal nations, even when there is broad agreement on research goals and objectives. University open research policies that do not explicitly address Indigenous sovereignty fall short of the open research principles they intend to support and should be revised. Formal adoption of principles for ethical research with sovereign tribal governments by universities is needed to improve coordination and trust among university and tribal researchers and members.

American Indian and Alaskan Native tribes and Native Hawaiian and Pacific Islander communities (hereafter referred to as “Indigenous communities”) have rights as sovereign entities to exercise control and ownership over all data and information generated by their tribal nation, members, resources, or land.^1^ Universities have “openness in research” policies that assert control or ownership of data collected by university researchers to ensure the universities’ ability to publish findings freely.^2^ These two principles, data sovereignty and openness in research, can create challenges to collaboration between university researchers and Indigenous communities. Here, we describe our recent study in which this occurred and summarize its resolution. Based on our case analysis, we affirm that U.S. research institutions that wish to conduct research in partnership with Indigenous sovereign nations must respect tribal data ownership policy, regardless of university institutional policy. We found that university open research policies that do not explicitly address Indigenous sovereignty fall short of the research principles they intend to support and need revision.

This case analysis is based on our University of Minnesota (UMN) PhD-level graduate student study of regional cultural ecosystem services, defined as the intangible benefits that arise from the interaction of people with the environment.^3^ The objective of the research was to examine how social factors including sociodemographics, personal and social identity, and social context influence cultural ecosystem services associated with local surface waters (lakes, rivers, and streams) to inform equitable natural resource management. The approach included a survey of a purposive sample of community members about their experiences related to cultural ecosystem services, followed by semi-structured qualitative interviews with a subset of survey participants with diverse backgrounds. The study area was the St. Louis River estuary region, located in the traditional and contemporary lands of the Anishinaabe people, also known as Ojibwe or Chippewa.^4^ The area included the reservation of a local federally recognized band of Ojibwe. The project aimed to include local Native American residents as one sociodemographic in the regional study; the focus of the study was not specifically on Native American cultural ecosystem services. At the time of publication of this article, data analysis and reporting is ongoing.

The five of us identify as White scientists and educators with training and work experience in institutional settings established based on Western philosophies, and we have a cumulative 96 years of experience working in the study area. We frame our discussion within the paradigm of institutional science to address the question of how institutions could better engage in sustained and mutually beneficial partnership with Indigenous communities. We endeavor to include accurate and vetted representations of Indigenous perspectives and scholarship.

## INDIGENOUS RESEARCH AND DATA SOVEREIGNTY

In 2007, the United Nations Declaration on the Rights of Indigenous Peoples (UNDRIP), supported by the U.S., affirmed that Indigenous peoples have inherent rights to self-determination. These rights include the right to “maintain, control, protect, and develop their cultural heritage, traditional knowledge, and traditional cultural expressions, as well as the manifestations of their sciences, technologies, seeds, medicines, knowledge of properties of flora and fauna, sports and traditional games, and visual and performing arts. They also have the right to maintain, control, protect and develop their intellectual property over such cultural heritage, traditional knowledge, and traditional cultural expressions.”^5^

There are currently 574 federally recognized tribes in the U.S.^6^ Although the UNDRIP grants all Indigenous peoples inherent rights to self-determination, there are approximately 400 nonfederally recognized Indigenous tribes in the U.S.,^7^ and the Indigenous communities in Puerto Rico, Guam, American Samoa, the Virgin Islands, the Northern Mariana Islands, the Republic of the Marshall Islands, the Federated States of Micronesia, and the Republic of Palau are also not federally recognized.^8^ Although tribal sovereignty in the U.S. was affirmed in *Worcester v. Georgia*^9^ and despite continual Indigenous resistance, history abounds with examples of theft of lands; genocide; forced relocation, sterilization, and assimilation; and other grave harms perpetuated by colonial governments and federal policies.^10^ These harms have had lasting effects on individuals and communities including generational trauma and loss of Indigenous languages, cultures, and lands.^11^

“Research sovereignty” refers to the right of Indigenous communities to set standards for research conduct and to expect equal participation in research.^12^ Data sovereignty includes “the right to govern the collection, ownership, and application of data about Indigenous communities, peoples, lands, and resources.”^13^ Examples of the abrogation of research and data sovereignty include medical experimentation on tribal members without consent^14^ and exploitation of tribal knowledge for private-sector benefit.^15^ Research relationships in the past have often been based on subjugation, fraught with White saviorism, and lacked reciprocity or respect for cultural norms.^16^ Research design often included little or no involvement from Indigenous communities and little understanding of or concern for community priorities, values, or protocols.^17^ Research reporting has included stereotyping, publication of sensitive materials without consent, and inadequate reporting back to the community.^18^ These examples suggest the critical importance of research sovereignty to Indigenous communities.

In the U.S., the Common Rule, the federal policy for the protection of human subjects in research,^19^ has helped address some of these issues by protecting individuals from harms due to research. However, issues persist. For example, as recently as the 1990s, researchers at Arizona State University took blood samples from members of the Havasupai tribe in Arizona with consent for use in a genetic study of diabetes.^20^ Researchers shared the samples with other universities and used them in additional published studies about the tribe without the knowledge or consent of individuals or the Havasupai tribe. Blood holds cultural significance for the Havasupai, who found that these additional studies were harmful, stigmatizing, and culturally offensive.^21^

The Common Rule explicitly protects individuals but may not adequately protect groups or communities from harm.^22^ This emphasis on individual protections is consistent with Western philosophies, but Western and Indigenous epistemologies and methodologies have different assumptions, values, beliefs, rules, and cognitive structures.^23^ Although each Indigenous community is unique, Indigenous worldviews often value holistic approaches over the isolation of system components, whereas Western science tends to be reductionist and based on testing hypotheses and isolating effects.^24^ Unlike Western worldviews, Indigenous knowledge is often cocreated, shared among a group, and held by knowledge custodians.^25^ Indigenous cultures often value group identity, which contrasts with the Western emphasis on individual identity, autonomy, and property ownership.^26^ In the absence of a national ethics policy for conducting research involving Indigenous peoples that protects their communities, Díaz Ríos et al. assert that research and data sovereignty are “a right, but [in practice are] still an aspiration for many Indigenous peoples.”^27^ Therefore, Indigenous communities can exercise research and data sovereignty to protect themselves from harms.^28^

Indigenous communities have taken action to protect their research and data sovereignty by establishing their own institutional review boards (IRBs).^29^ In 1996, the Navajo Nation of the Southwestern U.S. was the first to establish a tribal IRB.^30^ Currently, there are 15 independent tribal IRBs in the U.S.^31^ For federally recognized tribes without IRBs, human subjects research is reviewed by regional Indian Health Service (IHS) IRBs.^32^ Because tribal IRBs aim to protect both individuals and communities, they can have requirements that go beyond federal requirements in the Common Rule. For example, the Tribal Research Code of the Ho-Chunk Nation (Wisconsin) applies to all research “conducted within the Nation’s Territory, whether involving human subjects or not, and all research regarding materials wherever … the Nation has a claim of intellectual, cultural or other ownership, legal or equitable.”^33^ In addition, many tribal and IHS IRBs also require prepublication review of research results.^34^

## CASE ANALYSIS

**Figure 1.**
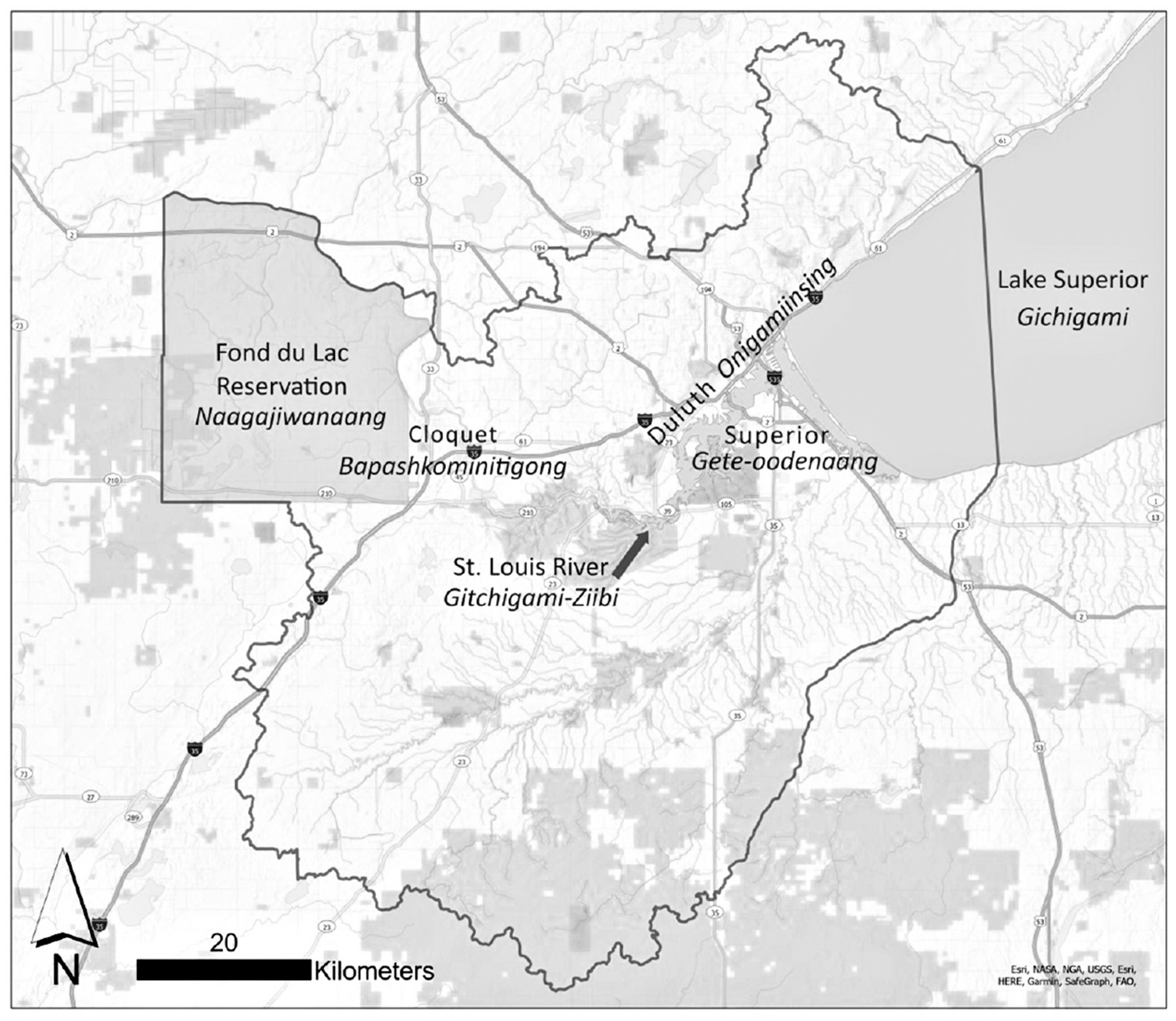
Map of Study Area The study area is the St. Louis River estuary of Lake Superior and includes the communities of Duluth and Cloquet, Minnesota, and Superior, Wisconsin. Ojibwe place names are shown in italics.^1^ ^1^ “Gidakiiminaan (Our Earth) Atlas” Great Lakes Indian Fish and Wildlife Commission, 2007, https://glifwc.org/publications/pdf/Atlas.pdf.

The St. Louis River estuary of Lake Superior (Nagaajiwanaang) includes the lower 39 miles of the St. Louis River and the immediate watershed downstream (see figure 1). Our study area largely coincides with the St. Louis River Area of Concern (AOC), a designation by the U.S. Environmental Protection Agency (EPA) for areas with a legacy of industrial contamination and habitat loss.^35^ This AOC has received at least 87 million federal dollars to address impairments in designated uses from 2010-2021.^36^ Although the AOC program is focused on restoring beneficial uses of waterways, limited assessment has been done to evaluate the impacts of these investments on communities, and it is unclear if the benefits of the investments are socially equitable.^37^ The research objective of our case study was to examine community impacts and equitability of AOC and related investments through cultural ecosystem service assessment.

The study area included the communities of Duluth (Onigamiinsing in the Ojibwe language) and Cloquet (Bapashkominitigong), Minnesota; Superior (Gete-oodenaang), Wisconsin; and several additional townships situated on the St. Louis River (Gitchigami-Ziibi) estuary, at the eastern end of Lake Superior (Gichigami). The study area is in the traditional and contemporary lands of the Anishinaabe people, which includes the reservation lands of a local Ojibwe Band (hereafter referred to as “the Band”), and is situated within the 1854 and 1842 Ceded Territories.^38^ People who self-identify as “American Indian or Alaska Native” constitute 2.5% of the population of the three major towns in the study area.^39^ The Band is a partner in ongoing restoration activities in the St. Louis River estuary and, along with other projects, is leading a major effort to restore manoomin (wild rice), an annual aquatic grass and a sacred food and medicine vital to Ojibwe lifeways, food sovereignty, and cultural identity.

Our study was situated in an historical and contemporary context of fraught relationships between Indigenous communities and research institutions. Minnesota has 11 federally recognized tribes and eight nonrecognized Indigenous communities.^40^ UMN has a current initiative that aims to understand the history of its relationship with these communities.^41^ This includes unethical medical research on impetigo and nephritis conducted during the Cold War on children of the Red Lake Band of Lake Superior Chippewa Reservation in Minnesota.^42^ A recent controversy involves the study of manoomin, a plant sacred to the Ojibwe. UMN has been conducting research on manoomin since 1950, including developing domesticated varieties and, in 2000, mapping the genome.^43^ This research was done without consultation with, compensation to, or acknowledgement of tribes who have for centuries been responsible for the stewardship and management of wild rice.^44^ The six bands of Minnesota Chippewa (Ojibwe) vehemently oppose this research because cross-contamination could change the genetic nature of natural manoomin stands, which could violate retained treaty rights to traditional, ceremonial, and subsistence relationships with the sacred plant.^45^ Chairman Goggleye Jr. of the Leech Lake Band explained it this way: “The Creator has given us many things. Every time we try to change [what we are given], it messes things up. I’m afraid this will happen to our wild rice beds. To [genetically engineer] wild rice would be disrespectful to the First People who inhabited this land . … It would be morally wrong.”^46^ Further, the ownership of genetic material, or claims to copyright in perpetuity, of a plant resource (especially by non-Indigenous peoples) is inconceivable to Ojibwe people. In a letter to the university, President Norman Deschampe of the Minnesota Chippewa Tribe asserted, “We object to the exploitation of our wild rice for pecuniary gain. We are of the opinion that the wild rice rights assured by treaty accrue not only to individual grains of rice, but to the very essence of the resource. We were not promised just any wild rice; that promise could be kept by delivering sacks of grain to our members each year. We were promised the rice that grew in the waters of our people, and all the value that rice holds.”^47^ For these reasons, Minnesota Ojibwe governments called for a moratorium on the wild rice breeding program and for protection of their intellectual property rights to wild rice in 2003.^48^ Despite this request, wild rice genetic research at UMN is ongoing. However, a recent collaboration between UMN researchers and regional tribes to study regional manoomin declines has sought to begin addressing some of these past harms.^49^

Our study was subject to review by the Band’s tribal IRB because it would include data collection on the reservation from self-identified Native Americans (information about tribal affiliation or descent was not collected). Their tribal IRB was established in 2005,^50^ and recommends approval or denial of all human subjects research to the main governing body, the Reservation Business Committee (RBC), which has the authority to make a final decision.^51^ All human subjects research conducted within the jurisdiction of the Band must be reviewed by the tribal IRB regardless of review by other IRBs. The guiding principles for the tribal IRB policy are the Seven Grandfather Teachings of the Band and the *Belmont Report*, the 1978 report by the National Commission for the Protection of Human Subjects, which outlines the fundamental ethical principles to protect human subjects.^52^ All research dissemination materials must be reviewed and approved by the tribal IRB and the RBC. The Band “reserves the right to recommend edits, negotiate changes, and if needed, to deny publication or research dissemination regarding IRB-approved research that it considers to be harmful, or potentially harmful, to individuals, tribal communities, [Band] resources, or the Tribe.”^53^ With respect to data ownership, the tribal IRB policy states that the Band “shall retain all ownership, property, trademark, copyright, and other rights to cultural, linguistic, and historic information that is not the intellectual property of the Researcher.”^54^ The policy does not explicitly state that a formal research and data-sharing agreement between the tribe and researchers is required, but it is implied by the data ownership policy.

## RESEARCH ENGAGEMENT WITH INDIGENOUS COMMUNITIES

To protect Indigenous communities, Indigenous scholars recommend decolonization of research practices, including employing community-engaged research, Indigenous epistemologies and methodologies, and leadership by Indigenous researchers.^55^ In our case, funding and academic constraints required this research be led by university researchers. This study was designed as part of a university PhD degree program’s requirements consistent with the dominant practices and expectations of institutional science. Furthermore, the study included research objectives and encompassed a population larger than the Indigenous community.

The likelihood of research findings resulting in new knowledge that can benefit the Indigenous community ultimately depends on the community’s engagement and tribal oversight. The local Band’s Resource Management Division (RMD) expressed support for our study and provided feedback during the study design stage. The RMD water quality manager helped articulate to the tribal IRB and RBC the potential benefits of the research. We convened a community advisory group of 11 community representatives including Indigenous representatives to solicit input on the study design and participant recruitment. In response to the initial tribal IRB review described below, we also convened a second advisory group that consisted of five Ojibwe representatives to provide input on the study design from an Indigenous perspective. In addition, individual consultation was done between the first author and many additional stakeholders representing community organizations and government agencies in one-on-one meetings.

We identified ways that the Band might benefit from this study through discussions with the Band’s RMD and members of the Indigenous advisory group. This included that the research has the potential to help non-Indigenous natural resource managers understand how Indigenous members of the community experience cultural ecosystem services in the context of their identity, worldview, and culture, and which cultural ecosystem services are important to those who self-identify as Native American. The knowledge that comes from this research could be applied in the design and evaluation of projects to help remove beneficial use impairments in the St. Louis River AOC. Our research might help negotiations for Natural Resource Damage Assessment for a local Superfund site in a tribally important area, for the restoration and protection of culturally important manoomin beds, and for the design of climate adaptation projects. Further, our results could help inform the evaluation of the equitability of outcomes of restoration and protection efforts in the AOC.

**Figure 2.**
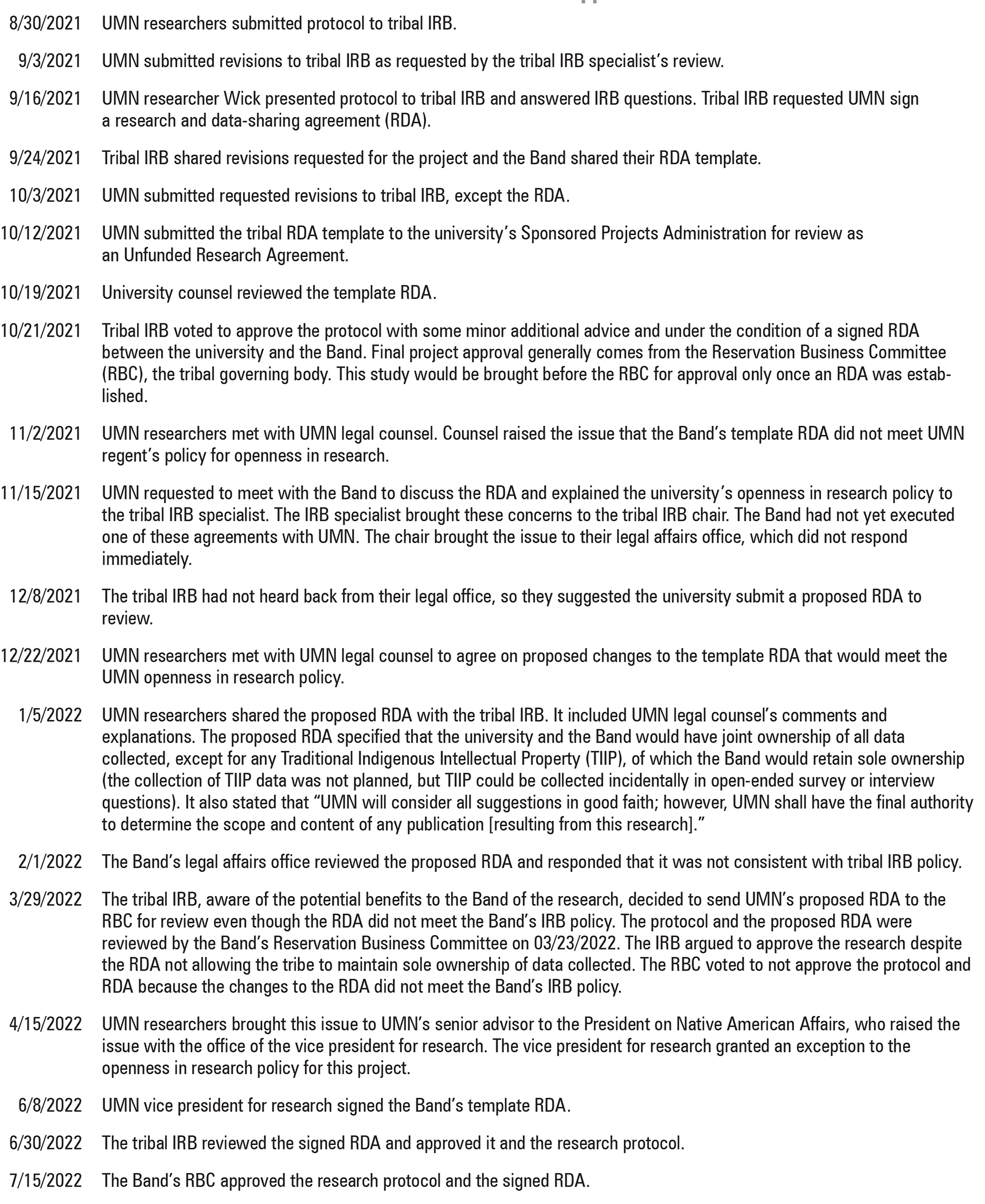
Timeline of Tribal IRB Review and Approval Process

We submitted the research protocol summarizing the study design and human subjects protections to the tribal IRB on August 30, 2021 (see figure 2 for a timeline of our protocol). The protocol was reviewed by the tribal IRB within a month of submission. During review, the Band requested protocol revisions including engagement of an Indigenous advisory group to review the survey and recruitment materials to ensure they would appropriately capture cultural ecosystem services for Indigenous people. The tribal IRB requested changes to the survey participant consent form. They also asked the university to sign a research and data-sharing agreement (RDA), for which the tribal IRB shared a template. After revisions were submitted in October 2021, the tribal IRB approved the protocol. At that time, university counsel in the UMN Office of the General Counsel reviewed the RDA template offered by the Band. Consistent with tribal IRB policy, the template stated that the Band would maintain sole ownership of all data collected on the reservation, or in this case, for any respondent who self-identified as Native American. Researchers and funders could use these data provided they maintained confidentiality and obtained approval from the tribal IRB prior to publication or research dissemination. The university’s legal counsel interpreted these terms of the agreement as impinging on UMN’s “Openness in Research” policy, which states that the university “shall not accept restrictions on participation in University research or on the dissemination of the results of University research,” and that the university “reserves the right to publish and present research results, individually and in collaboration with other researchers.”^56^

To address this issue, we requested a meeting with the tribal IRB chair or Band’s legal affairs office to understand the Band’s concerns and attempt to find a solution. The Band was unable to accommodate this request in a timely manner and suggested that UMN submit an RDA proposal reflecting their requirements. Working with the university’s legal counsel, we drafted a proposed RDA specifying that the university and the Band would have joint ownership of all data collected, except for any Traditional Indigenous Intellectual Property (TIIP), of which the Band would retain sole ownership (the collection of TIIP data was not planned, but TIIP could be collected incidentally in open-ended survey or interview questions). The proposed RDA, consistent with UMN’s openness in research policy, stated that “UMN will consider all suggestions in good faith; however, UMN shall have the final authority to determine the scope and content of any publication [resulting from this research].”

We submitted this proposed RDA to the tribal IRB in January 2022. The Band’s Legal Affairs office reviewed the proposed RDA and responded that it was not consistent with the Band’s IRB policy.^57^ The tribal IRB, aware of the potential benefits to the Band of this research, argued to the RBC that the research should be approved despite the proposed RDA not allowing the Band to maintain sole ownership of data collected. However, the RBC disagreed and did not approve the university’s RDA proposal.

At that time, we engaged the senior advisor to the university president on Native American Affairs. The university created this position in 2021 to “acknowledge the historic injustices against Native Americans in Minnesota.”^58^ The senior advisor brought the matter to the attention of the university’s vice president for research, who indicated that his office could review specific circumstances and grant waivers to the openness in research policy. Through previous discussions regarding tribal sovereignty and partnerships, the vice president for research was familiar with concepts of tribal data sovereignty. After review of our proposed research and the tribal requirements, the vice president for research granted a waiver to the openness in research policy for this study and signed the tribe’s template RDA. Thereafter, the tribal IRB and the RBC approved the RDA. From research protocol submission to final RBC approval, this process took 11.5 months.

## DISCUSSION

Open research policies, Indigenous sovereignty, and human subjects protections must be integrated when designing and implementing research involving Indigenous communities, tribal nations, or their resources. Our experience in this case should reinforce for university researchers that university policies do not supersede tribal sovereignty over data, knowledge, or property.

UMN and many other research universities’ openness in research policies do not currently include considerations for tribal sovereignty.^59^ Based on our case analysis, we recommend revising these policies to include explicit exceptions for research partnerships with sovereign tribal nations and Indigenous communities to facilitate research partnerships and protect Indigenous communities. UMN’s openness in research policy is designed to protect research from corporate influence exerted through research funding. This is a serious concern. The fundamental purpose of universities, to promote the advancement of society through knowledge, depends on academic freedom, or the right to teach and conduct research without censorship.^60^ However, as Brugge and Missaghian point out, concern for influence exerted through research funding is not generally applicable to Indigenous nations, who rarely fund university research.^61^

Government funding agencies, which universities largely rely on for funding, increasingly have open science requirements to ensure publicly funded research samples, data, software, and publications are openly available, and for the purpose of supporting open research.^62^ These policies generally reflect the FAIR principles of Findability, Accessibility, Interoperability, and Reuse of digital assets.^63^ The Global Indigenous Data Alliance developed the CARE principles to complement the FAIR principles and protect Indigenous data sovereignty. CARE stands for Collective benefit, Authority to control (data, resources, and research by Indigenous communities), Responsibility (of researchers working with Indigenous data), and Ethics (to prioritize Indigenous communities’ rights and well-being).^64^ Although these principles have yet to be formally adopted in the U.S., federal funding agencies are increasingly acknowledging the need to protect Indigenous research sovereignty.^65^ Federal policies fall under the purview of the federal trust responsibility with federally recognized tribes, the obligation of the U.S. government to secure tribes’ rights to self-governance.^66^ Therefore, explicit exceptions to open research policies for tribal sovereignty would be consistent with funding agency policies and federal trust responsibilities.

In this case, the local Band was unwilling to negotiate about data ownership, leaving UMN with two main options consistent with tribal sovereignty: to accept the Band’s template RDA, or to abandon the aspects of the research involving the Band and its members and resources. Inefficient coordination between the university and tribal government, due to UMN’s omission of recognition of Indigenous sovereignty in its openness in research policy, significantly delayed this PhD research project. Short of preventing the research, administrative delays resulting from protracted research policy negotiations among parties, or internally within the university, have been interpreted as infringing on academic freedom and can be a hindrance to postgraduate studies.^67^

Preventing, halting, or interfering with research that stands to benefit Indigenous communities also contradicts recent interpretations of the Common Rule. Freeman argued that the principle of justice in the *Belmont Report*, which underpins the Common Rule, should be applied not just to individuals but also to communities.^68^ Such communities should not be asked to participate in research with little benefit to them, and conversely, should be included in research that is potentially of benefit to them. A 2021 recommendation from the Secretary’s Advisory Committee on Human Research Protections to the Secretary of Health and Human Services reiterated this, stating that due to the systemic exclusion of certain populations from research, justice was lacking in the Common Rule.^69^ This interpretation of the Common Rule implies an ethical obligation to include (and for universities to facilitate inclusion of) Indigenous communities in university research from which they would benefit. For example, the exclusion of Indigenous participants in this study would inhibit potential benefits like improving cross-cultural understanding of plural values associated with nature, and increasing the equitability of the environmental restoration outcomes for Indigenous communities and individuals.

Under the terms of the RDA signed by the local Band and UMN, the Band retains the right to approve or not approve publication of research from study research “that it considers to be harmful, or potentially harmful, to individuals, tribal communities, […] resources, or the Tribe.” This is prima facie inconsistent with university academic freedom doctrines, but it reflects the special case of a research partnership between the university and a sovereign nation. The practical implications of the RDA will depend on the specific research. In the case of our study, we think it unlikely that the Band would prevent all publication of our research findings because of the relatively benign nature of the data collected (e.g., perceptions of cultural ecosystem services rather than medical information). Furthermore, we expect tribal review and comment on research results to contribute valuable perspective and context, while also providing a final check on inappropriate dissemination of tribal knowledge to prevent harms from occurring. However, the risk of tribal lack of approval of research products must be a consideration in the original conception and design of research. Tribal IRB review and input on research design, along with community input, such as what we received from the Indigenous advisory group, may also help diminish the risk of lack of approval.

The UMN IRB and the tribal IRB both reviewed the research protocol for this study. At the time of our proposed study, the UMN IRB had not previously worked with the local Band’s IRB and was unfamiliar with their requirements and process. Navigating study review by multiple IRBs can be inefficient,^70^ and IRBs are often reluctant to cede review to another IRB through reliance agreements.^71^ Improving cooperation between university and tribal IRBs with overlapping jurisdictions would reduce administrative burdens on researchers. This could include training university IRB staff on tribal IRB requirements so they could provide technical and legal guidance to researchers. Because tribal IRBs often require more information and may have different requirements than standard IRBs,^72^ process flexibility by university IRBs would ultimately help support tribally engaged research. Such changes would be consistent with the flexibility of review requirements allowed by the Common Rule to accommodate local context.^73^

University compliance with tribal ethics review can help address researcher and systemic biases with respect to Indigenous communities or partners.^74^ This is especially important in qualitative studies set in Indigenous communities and is a central tenant of rigorous social science research.^75^ Like Angal et al., we found that the tribal IRB review was a mutually beneficial “mechanism of independent community oversight” rather than a “regulatory hurdle.”^76^ We received constructive feedback from the tribal IRB and from the Indigenous advisory group that the tribal IRB requested be convened, which improved our study design and the likelihood of collecting reliable data. The advisory group provided study design oversight that improved the cultural sensitivity of survey and interview questions while maintaining scientific integrity. These changes, along with tribal IRB approval, which helps ensure study participants that the research has direct oversight from the Band, likely improved participant recruitment.^77^

Requiring us to have tribal review of manuscripts provides an additional level of oversight to ensure that results are interpreted appropriately in the context of Ojibwe culture and worldviews. Throughout our study, and despite university-driven efforts to “acknowledge the historic injustices”^78^ and improve tribal-university relations, we found a lack of awareness and understanding within the university’s legal and sponsored program offices about tribal sovereignty or university relations with Indigenous communities or nations. We found that these high-level initiatives had not yet “trickled down” into university operations, and the university’s actions with respect to tribal affairs were not consistent across programs. This is not an isolated problem. Turpel-Lafond and Chondoma identified this as a significant barrier to partnerships, stating, “Institutions of learning must get their own houses in order in significant ways to engage with [Indigenous] individuals and Nations appropriately.”^79^ Wong et al. called for researchers to obtain an understanding of not just the sociopolitical landscape around their research sites, but also of Indigenous peoples’ rights to and manifestations of self-determination.^80^ University-sponsored training for researchers and graduate students conducting or hoping to conduct research in partnership with Indigenous communities has been very limited.^81^ However, to help address this need, the UMN Office of Native American Affairs recently established guidance for researchers wishing to conduct research with Indigenous communities.^82^ These efforts as well as the work of Indigenous scholars within universities may help shift university culture and cultivate political capital for changes in university policy that will support research partnerships with Indigenous communities in the future.

## CONCLUSION

This case analysis identified a major challenge with respect to university research partnerships with Indigenous communities, which is the lack of explicit considerations for Indigenous research and data sovereignty in university open research policies compounded by an incomplete understanding within the university of tribal sovereignty. In the case described herein, this resulted in a significant delay in research progress and increased administrative burden on the part of the Band and the university. To support university research that is inclusive of and potentially beneficial to Indigenous communities, we recommend universities undertake efforts to educate and provide guidance for researchers and staff on tribal rights to self-determination and research sovereignty. Further, we recommend universities include explicit, proactive considerations for Indigenous research and data sovereignty in academic freedom and research policies. Policies should be developed in consultation with Indigenous governments and advisors. To facilitate this, resources need to be adequately allocated not only to university research and ethics review programs but also to tribal IRBs and research advisors. These changes will facilitate ethical collaboration among universities and Indigenous communities and ultimately lead to mutually beneficial outcomes consistent with Indigenous sovereignty.
